# Design of a Facile Antifouling Sensor Based on the Synergy between an Antibody and Phase-Transited BSA

**DOI:** 10.3390/bios13121004

**Published:** 2023-11-29

**Authors:** Siqi Wang, Xinru Dong, Jialu Li, Jialei Liu, Yifei Ruan, Yinqiang Xia

**Affiliations:** 1 College of Chemistry and Pharmacy, Northwest A&F University, Yangling 712100, China; wangsiqi@nwafu.edu.cn; 2 College of Food Science and Engineering, Northwest A&F University, Yangling 712100, China; dxr1011@nwafu.edu.cn (X.D.); li_jialu@nwafu.edu.cn (J.L.); liujialei@nwafu.edu.cn (J.L.); ruanyifei@nwafu.edu.cn (Y.R.)

**Keywords:** SPR, antifouling, phase-transited BSA, antibody synergy

## Abstract

Nonspecific adsorption has always been a critical challenge for sensor detection; thus, an efficient and facile approach for fabricating antifouling sensors is highly desirable. Here, we developed an antifouling coating on sensor surfaces, conveniently made with a simple drip of phase-transited BSA (PTB) followed by a modification with a peanut allergen antibody, which unexpectedly provides synergistic antifouling properties in sensors. Atomic force microscopy and scanning electron microscopy were used to evaluate the surface evenness. Optimizations in terms of PTB modification time and concentrations were performed using surface plasmon resonance by measuring protein resistance capabilities. Compared to bare Au surfaces, the PTB-modified surfaces exhibited low adsorption against BSA (<10 ng/cm^2^) and good resistance against lysozyme (Lyz). After immobilizing antibodies, the antifouling performance of the sensor coatings had an obvious enhancement, with almost no BSA adsorption and low lysozyme adsorption. The target recognition was also analyzed to verify the good sensing performance of the antifouling sensor. This understanding of antibody synergy provides suggestions for the development of antifouling sensors.

## 1. Introduction

Biosensors are a kind of highly sensitive and efficient analytical technology that has been widely used in many fields, including clinical diagnosis [1], environmental monitoring [2], and food safety [3]. As a typical analytical biosensor, surface plasmon resonance (SPR) is employed to measure the refractive index changes on the sensor surface and is used to monitor the molecular interaction between analytes and the surface-modified ligands (e.g., enzymes, antibodies, and aptamers, etc.) [4,5]. Due to the advantages of high sensitivity, free labels, and real-time detection, SPR is commonly applied for trace analyte detection [6]. However, the sample matrix interference caused by the nonspecific adsorption of complex media like food and serum can significantly disturb the SPR response to the analyte binding, which negatively affects the sensitivity and accuracy of the SPR sensor during trace detection [7,8]. Though sample pretreatment could solve this problem to some extent, the process is complicated and time-consuming [9]; therefore, the development of antifouling surfaces against nonspecific adsorption is of great significance for SPR detections.

Over the decades, considerable efforts have been devoted to fabricating effective antifouling coatings [10,11,12]. Poly(ethylene glycol) (PEG) and its derivatives have long been treated as important antifouling candidates for preventing fouling adsorptions on sensor substrates [13,14,15]. However, their disadvantages of oxidative degradation, poor water solubility, and limited fouling resistance cannot be ignored [16]. Dextran has been commercialized to form antifouling coatings for SPR sensors, but it is still a challenge against complex samples [8]. Zwitterionic materials, as excellent alternatives to PEG, have drawn great attention due to their better stability and antifouling performance. With a balance of positive and negative charges, zwitterionic polymers such as poly-(carboxybetaine methacrylate), poly(2-methacryloyloxyethyl phosphorylcholine), and poly(sulfobetaine methacrylate) could provide more strongly bound hydration layers than PEG coatings, contributing to a repulsive effect against the approach of foulants [17,18,19,20]. Meanwhile, their overall charge neutrality provides a further benefit by reducing the electrostatic interactions of the foulants on the modified coating’s adsorption [21]. Unfortunately, the complex synthetic process of zwitterionic materials limits their widespread application in sensors.

Bovine serum albumin (BSA) has always been deemed an ideal molecule to reduce the interference of nonspecific background in ELISA and Western blotting assays [22]. Though BSA has been cross-linked to create an antifouling electrochemical sensor, it is still rarely used in the construction of antifouling surfaces for SPR sensors [23]. The reason may be due to the weak physical adsorption of BSA on the sensors. Interestingly, the phase-transited BSA (PTB) that can be obtained through a reduction in disulfide bonds has been proven to possess the capability of stable adhesion, as well as good antifouling performance [21,24]. Without any pretreatment, the PTB can easily and stably adsorb on multiple types of substrates and shows an efficient resistance against contaminants, providing an impressive foundation for the construction of antifouling sensors [25,26]. Additionally, abundant groups of BSA could be activated to immobilize recognition ligands to establish specific sensors, though the introduction of ligands may affect the surface structure and the corresponding antifouling performance of the sensors [27]. However, few studies have been conducted on the specific impact of the antifouling capability of modified coatings. Some differentiated effects appeared on the fouling-resistance capabilities of different surfaces when the antibodies were modified on these surfaces [28]. An antibody, as a kind of recognition molecule, is essentially a protein. Just like BSA, we wondered if the immobilization of antibodies on sensor surfaces could also be involved in the antifouling process.

**Scheme 1:**
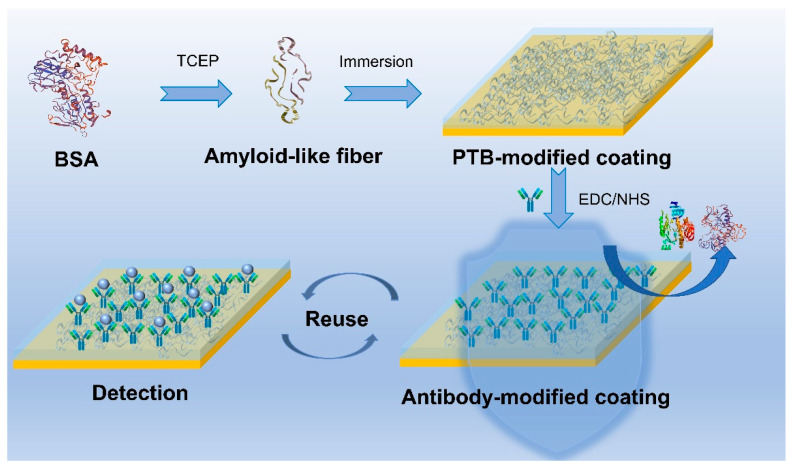
Schematic for the preparation process of facile antifouling sensors.

In this work, we developed a facile antifouling sensor with coupling specific antibodies on phase-transited BSA (Scheme 1). The PTB coating could be rapidly formed by simple drips to provide an antifouling substrate, as well as abundant active groups for further modifications, on sensor chips. An antibody against full peanut allergens was selected to be immobilized on the sensors. The antifouling performance of bare surfaces, PTB-modified surfaces, and antibody coatings were then analyzed to verify the effect of the antibody on the resistance capability of the sensors against the nonspecific adsorption of contaminants. Finally, a simple detection was performed to evaluate the analytical performance of the sensors.

## 2. Experimental Section

### 2.1. Materials

Bovine serum albumin (BSA), tris(2-carboxyethyl)phosphine (TCEP), lysozyme, ethanolamine, Thioflavin T (ThT), N-Hydroxysuccinimide (NHS), 1-(3-Dimethylaminopropyl)-3-ethyl carbodiimide hydrochloride (EDC), and Congo red were purchased from Aladdin Reagent Co., Ltd. (Shanghai, China). Phosphate-buffered saline (PBS, 0.01 M, pH 7.2–7.4) was purchased from Solarbio Reagent Co., Ltd. (Beijing, China). The peanut allergens and their corresponding antibody were obtained from Tianjin University of Science and Technology (the antigen consisted of all types of peanut allergens and the antibody was a monoclonal antibody from mice).

### 2.2. Modification of Phase-Transited BSA on Au Surfaces

Before the modification process, Au chips (BioNavis Ltd., Tampere, Finland) were cleaned by immersing them in alkali piranha solution (H_2_O/NH_3_·H_2_O/H_2_O_2_ = 5:1:1) at 75 °C for 15 min, followed by rinsing with ultrapure water. Then, the chips were placed in a plasma device for further cleaning for 20 min (Nanjing Suman Plasma Technology Co., Ltd., Nanjing, China). After being washed with ultrapure water and subsequently dried with N_2_, the chips could be used to fabricate antifouling surfaces. Specifically, BSA was mixed with TCEP (4 mg/mL) in a PBS buffer (10 mM pH 7.4) for a phase transition reaction. Different BSA concentrations of mixture solutions (0.5 mg/mL, 1 mg/mL, 2 mg/mL, and 4 mg/mL) were dropped on the chips to fabricate antifouling chips. Different incubation times (0.5 h, 1 h, 2 h, and 6 h) were examined to determine the optimum incubation time. Finally, after the chips were cleaned again with ultrapure water, the phase-transited BSA surfaces were ready for the next tests.

### 2.3. Morphology and Force Curve Measured by AFM

The surface morphology and force curves were measured in a tapping mode by an atomic force microscope. A silicon nitride (Si_3_N_4_) atomic force microscope probe with a force constant at 44.79 pN/nm and radius at 15 nm was selected. Before measurement, the chip samples were washed with ultrapure water and dried with N_2_. Each experiment was performed at room temperature at least three times.

### 2.4. Contact Angle Measurements

The water contact angles of bare Au and the phase-transited BSA-modified surfaces were measured by an SZ-CAM device (Sunzern Instrument Co., Ltd., Shanghai, China). Briefly, a 2 μL water droplet was deposited on the bare Au and modified surfaces. After they reached stability, a three-phase interface image was captured by a CCD camera. The corresponding contact angle could be easily calculated by fitting the profile of the droplet at the three-phase interface. Each peptide-modified surface was measured at at least three different positions to obtain the average angle, ensuring the repeatability of the measurements.

### 2.5. Measurements of Antifouling Performance on the Phase-Transited BSA Surface

To measure the nonspecific protein adsorption on the bare Au and modified surface, BSA and lysozyme were treated as contaminants and dissolved in PBS to 1 mg/mL for antifouling evaluation. An SPR instrument was used to determine the nonspecific adsorption by monitoring the real-time interaction between proteins and the modified chips. Briefly, an original baseline was first established using PBS buffer at a flow rate of 50 μL/min. Then, the contaminant, BSA, or lysozyme solution, was injected and flowed over the cell at a flow rate of 10 μL/min. The real-time adsorption process was observed within about 10 min. Following that, PBS buffer was used to rinse the cell again at 50 μL/min to obtain a new response signal line. Through analysis of the difference between the original baseline and final response signal line, the adsorption amount of contaminants could be precisely obtained with this simple process.

### 2.6. Fabrication and Evaluation of the Specific SPR Sensor

Briefly, the EDC (0.4 mol/L) and NHS (0.1 mol/L) solutions were mixed at a 1:1 *v*/*v* ratio, followed by flowing at a rate of 10 µL/min for 30 min to activate the carboxyl groups on the PTB-modified surface. Then, 10 μg/mL of antibody against peanut allergens was injected for immobilization at a rate of 5 µL/min for 30 min. Finally, the unreacted sites were deactivated by ethanolamine for 10 min. The antifouling performance of the antibody-modified sensors was measured in similar steps as above.

A simple immunoassay was designed for peanut allergen detection. Specifically, a series of concentrations of PA solutions were prepared in PBS buffer, and then the PA solutions were injected into the flow cell at a rate of 10 μL/min for x min to ensure an adequate binding with the modified antibody. Following that, 10 mM HCl was used to dissociate the antibody–antigen binding for the reuse of the sensor chips. All tests were performed at least three times for precise results.

## 3. Results and Discussion

### 3.1. Characterization of PTB-Modified Surfaces

**Figure 1:**
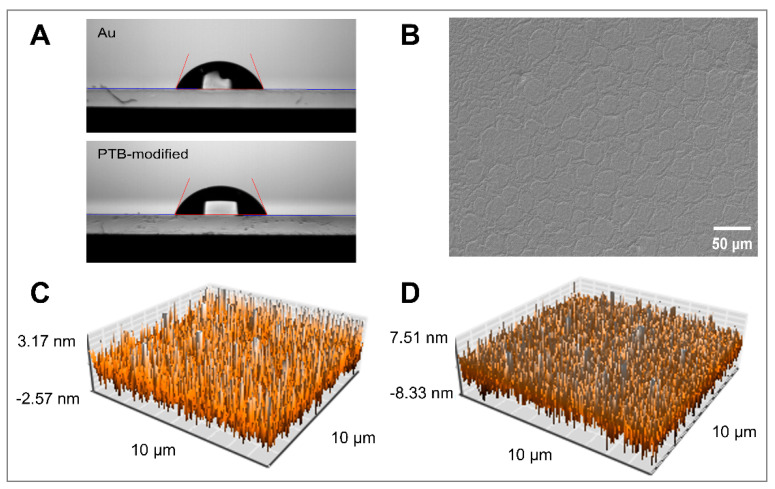
(**A**) Water contact angle of the bare Au and PTB-modified surface; (**B**) SEM image of uniform PTB film; and AFM images of bare Au (**C**) and the PTB-modified surface (**D**).

To understand the properties of the PTB coatings, surface wettability was investigated using a water contact angle instrument. As shown in Figure 1A, the water contact angle of the bare Au surface was 71.3° ± 1.7°. After being modified with PTB, the water contact angle had a slight decrease to 68.8° ± 1.5°, demonstrating that the modification of PTB did not change the hydropathy property of the sensor surfaces. Similar results were also obtained on silica surfaces (76.2° ± 2.7°), which met the results of other work [24]. Considering that the PTB modification was an offline process, fluorescent spectra were used to characterize the phase transition of BSA. After being mixed with TCEP, the disulfide bonds of BSA were reduced, which led to an increase in the β-sheet of BSA and induced BSA to form amyloid-like aggregations. ThT, as a kind of fluorescent indicator, can efficiently bind to amyloid structures and contribute to monitoring the occurrence of the BSA phase transition. As for native BSA, almost no fluorescent signals were observed. In contrast, the mixture of ThT and PTB showed a fluorescent signal at 484 nm, indicating the phase-transited process from native BSA to amyloid-like aggregations (Figure S1). Congo red was also used to demonstrate the fabrication of a PTB-modified surface. Due to the interaction between Congo red and the β-sheet structure, a uniformly colored film appeared after rinsing with water, which proved the formation of PTB film on the surface (Figure S2). The surface morphology was further investigated using SEM and AFM instruments. As shown in Figure 1B, a relatively uniform film was obtained by SEM scanning. With continuous irradiation of the high-power electron beam on the film for a few seconds, a hole in the film occurred, which also demonstrates the thin film fabrication of PTB (Figure S3). As for the AFM results (Figure 1C), the root-mean-square roughness (RMS) of bare Au was 1.1 ± 0.1 nm. After being modified with PTB, the RMS presented a little increase to 2.5 ± 0.2 nm with an increase in the thickness of 6.3 nm (Figure 1D), indicating that the PTB had assembled on the surface with equally distributed nanoscale asperities. All the results indicated that the PTB had been successfully modified on the surface.

### 3.2. Nonspecific Adsorption on Modified Surfaces

**Figure 2:**
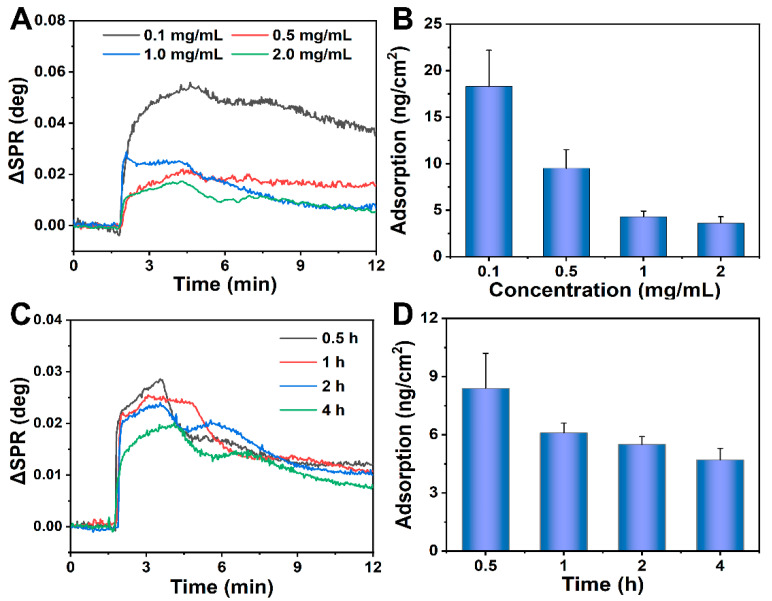
SPR spectrogram monitoring of PTB-modified surfaces with different concentrations against BSA adsorption (**A**) and statistic columns of the adsorption amounts for each PTB surface concentration (**B**). SPR spectrogram monitoring of PTB-modified surfaces with different incubation times against BSA adsorption (**C**) and statistic columns of the adsorption amounts for each PTB surface incubation time (**D**).

To evaluate the optimum antifouling performance, we employed SPR to monitor the interaction between contaminant proteins and PTB-modified surfaces. The core mechanism measures the amount of nonspecific protein adsorption on surfaces. BSA and lysozyme were used as model contaminant proteins to flow over the surfaces; corresponding SPR spectrograms were obtained and are shown in Figure 2. Due to the refractive index changes (BSA solution > PBS), the SPR response value increased sharply. As the adsorption proceeded, the rate of BSA adsorption tended to slow down. The dissociation process became dominant when PBS flowed again. The weakly adsorbed BSA could then be washed away until reaching the adsorption–dissociation equilibrium. With the real-time SPR response signals, the amount of BSA nonspecific adsorption could be easily read with high accuracy. The conversion ratio is that an SPR shift of 1° corresponds to a surface protein adsorption of 592.4 ng/cm^2^. Before the experiment, the PTB-modified surfaces were optimized by controlling the parameters of BSA concentration and incubation time. As shown in Figure 2A, a series of SPR spectrograms were recorded of the BSA nonspecific adsorption on PTB-modified surfaces with different BSA concentrations. On the 0.1 mg/mL PTB modified surface, the SPR response value changed after the adsorption of BSA at 0.036°. Compared to that of bare Au surfaces (0.291°), the SPR response dropped nearly eight-fold, suggesting that the PTB-modified surface exhibited effective resistance against protein adsorption (Figure S4). As the PTB concentrations increased to 0.5, 1, and 2 mg/mL, the SPR responses shifted to 0.016°, 0.007°, and 0.006°, respectively. Obviously, with the increase in PTB concentration, the response to BSA adsorption decreased further, demonstrating an improvement in antifouling performance. The reason may be that a higher BSA concentration contributes to a dense structure, which is beneficial in preventing BSA adsorption. According to the relationship between the SPR response signal and the mass of substrate adsorption on the surfaces, the mass amounts of BSA adsorption were obtained and displayed in the histogram. It was seen that the amount of BSA on PTB-modified surfaces was as low as 3.6 ng/cm^2^, which reaches the standard for ultralow adsorption (<5 ng/cm^2^) [29,30]. When the concentration exceeded 1 mg/mL, the resistance capability against BSA became slowly enhanced. A similar amount of BSA adsorption on PTB-modified surfaces of 1 mg/mL and 2 mg/mL were obtained at 4.3 ng/cm^2^ and 3.6 ng/cm^2^, respectively (Figure 2B). For the optimization of incubation time, 0.5, 1, 2, and 4 h were set to immobilize 1 mg/mL PTB on surfaces. As shown in Figure 2C, the SPR responses caused by BSA adsorption on all the PTB surfaces were captured, showing a minor range from 0.008° to 0.014° which indicates the weak effect of reaction time on resisting BSA adsorption. All the results presented a low adsorption level (<10 ng/cm^2^) and an almost 95% decline in BSA adsorption compared to that of bare Au. Regarding the prolonging of the incubation time, the PTB may fully react with the substrate, tending towards a relatively better antifouling performance. The average adsorption amounts for incubation times of 0.5 h, 1 h, 2 h, and 4 h were 8.3, 6.1, 5.5, and 4.7 ng/cm^2^, respectively (Figure 2D). When the reaction time exceeded 1 h, the improvement in BSA resistance became slower than that within 1 h. Meanwhile, compared to the amount of BSA adsorption of the overnight treatment (4.3 ng/cm^2^), that of the 4 h treatment was almost the same.

**Figure 3:**
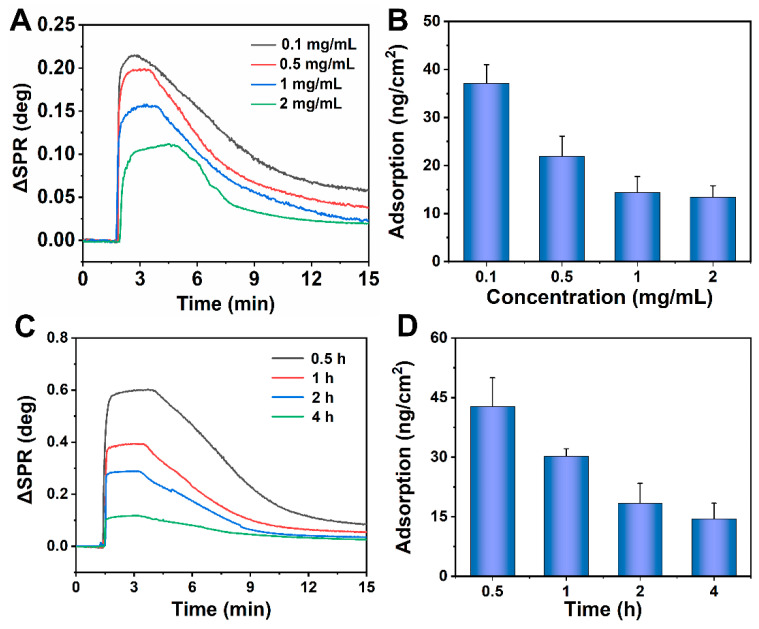
SPR spectrogram monitoring of PTB-modified surfaces with different concentrations against lysozyme adsorption (**A**) and statistic columns of the adsorption amounts of each PTB surface concentration (**B**). SPR spectrogram monitoring of PTB-modified surfaces with different incubation times against lysozyme adsorption (**C**) and statistic columns of the adsorption amounts for each PTB surface incubation time (**D**).

Similar results have also been observed for resistance against lysozyme. As shown in Figure 3A, the SPR response caused by lysozyme adsorption dropped with the rise in PTB concentration. Though the corresponding adsorption amounts of lysozyme decreased from 37.1 to 13.4 ng/cm^2^, the final adsorption amount of lysozyme was much higher than that of BSA when the PTB concentration was 2 mg/mL (Figure 3B). As for the prolongation in incubation time, the capability of lysozyme resistance also saw a significant improvement. The SPR response dropped more than three times, from 0.082° to 0.025° (Figure 3C), which is consistent with the results of BSA adsorption, demonstrating the importance of incubation time for the fabrication of PTB coatings. In contrast, there was an enhancement in lysozyme resistance (30.2 ng/cm^2^ of 1 h incubation and 18.4 ng/cm^2^ of 2 h incubation) when the incubation time increased from 1 h to 2 h, though it is not clear with BSA adsorption (Figure 3D). Compared to the adsorption level on bare Au surfaces (~132.6 ng/cm^2^, Figure S5), though the modified surfaces showed a certain degree of antifouling ability against lysozyme, the lysozyme adsorption was still much more obvious than with BSA. The reason may be attributed to the electrostatic interaction between the positively charged lysozyme and negatively charged BS; this also explains the ultra-low adsorption of BSA on the PTB-modified surfaces. The reduction in lysozyme adsorption in a more stringent buffer could also explain this phenomenon (Figure S6). Based on the antifouling results and the comprehensive consideration of the assembling efficiency of the modified surfaces, 2 h of incubation time and 1 mg/mL of PTB were chosen to be used in the following experiments.

### 3.3. Fabrication and Evaluation of Antifouling Sensor

**Figure 4:**
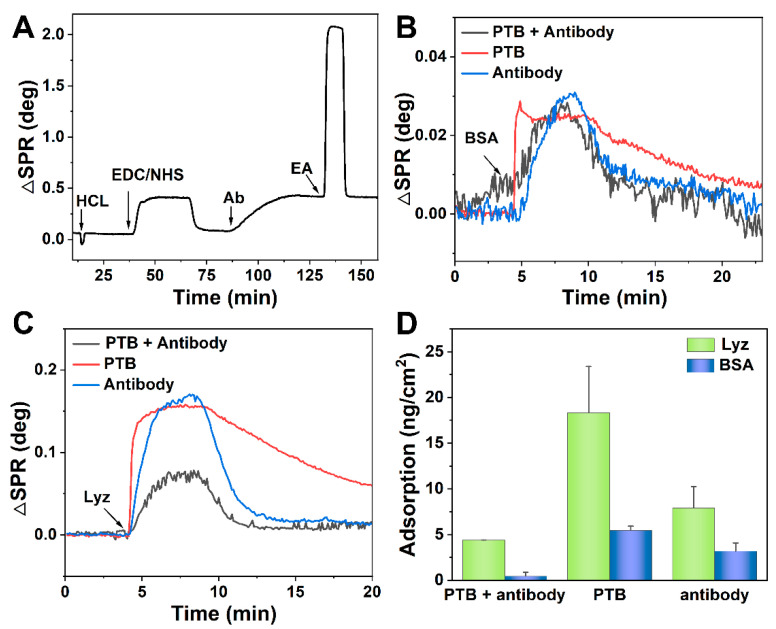
SPR spectrogram monitoring response shifts during the antifouling sensor modification process (**A**). SPR spectrogram of BSA (**B**) and lysozyme (**C**). Statistic column of the adsorption amounts of the modified antibody PTB, PTB, and modified antibody on the bare Au surface (**D**).

To fabricate an antifouling sensor, specific ligands can be immobilized on the PTB-modified surface for target detections. As shown in Figure 4A, the PTB-modified surfaces were activated by an EDC/NHS method. Before activating, the PTB surfaces were rinsed with 10 mM HCl to remove the weakly fixed PTB molecules. With the injection of the mixture of EDC/NHS, a sharp increase in SPR response occurred due to the changes in the refractive indexes. After rinsing with PBS, the SPR response decreased rapidly to a final value of 0.028°, which indicated that EDC/NHS had successfully activated the PTB surfaces. Then, 10 ng/mL of the antibody was injected into the cell at a slow rate to ensure an adequate reaction. The binding antibody induced an increase in the SPR response to a final value of 205.1 ng/cm^2^, demonstrating that the antibody was modified on the surfaces. After immobilizing, parts of carboxyl groups remain activated on the surface, which directly disturbs the specific recognition. Thus, ethanolamine was used to block the activated carboxyl groups. The nonspecific BSA adsorption was also used to evaluate the antifouling performance of the sensor surfaces. As shown in Figure 4B, the amount of BSA adsorption on the sensor surfaces (modified with PTB and antibody) was 0.46 ng/cm^2^, exhibiting a slight reduction compared to that of PTB surfaces.

The results of lysozyme adsorption also verified this phenomenon. The amount of lysozyme adsorption on the sensor surfaces was 4.39 ng/cm^2^, which is much lower than that on PTB surfaces. Unexpectedly, the introduction of antibodies showed no negative effect on protein resistance; on the contrary, it enhanced the antifouling performance of the sensor surfaces. To better understand this phenomenon, we first modified the antibody on the bare Au to observe if the antibody possesses antifouling capability. The antibody modification process is simple physical adsorption with the same concentration (10 μg/mL) as the immobilization on PTB surfaces. After the antibody was injected into the flow cell, the SPR response increased to 0.680°, demonstrating that the antibody had been successfully fixed on the surface. Following that, BSA and lysozyme were used to evaluate the fouling resistance capability of these antibody surfaces. As shown in Figure 4D, the amount of BSA adsorption was 3.17 ng/cm^2^. This adsorption amount is slightly lower than that of PTB surfaces, which reached a similar level as the reported PEG and hyaluronic acid surfaces that have always been used as antifouling materials [31,32]. As for the lysozyme, the adsorption amount lowered to 7.93 ng/cm^2^, surprisingly presenting a much better resistance capability against lysozyme than PTB surfaces. These results may be explained by the immobilization of antibodies on PTB-modified surfaces which contributes to the synergistic antifouling performance of sensor chips. Furthermore, the antibody potential was tested using a dynamic light scattering instrument. A weak negative charge from the antibody was obtained at −9.48 mV, which may contribute to shielding the electrostatic adsorption between the positively charged lysozyme and the sensor surfaces, as well as the negatively charged BSA.

**Figure 5:**
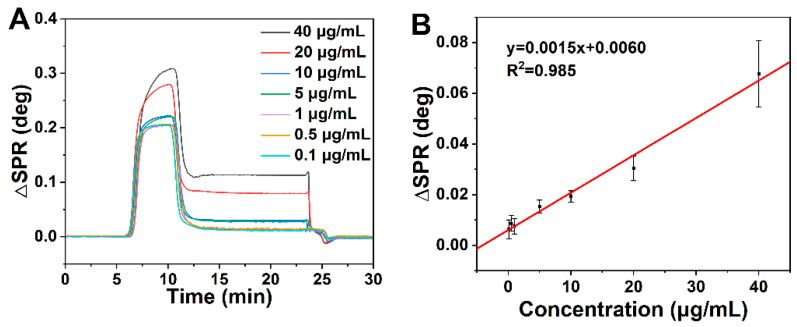
SPR response spectrogram (**A**) and standard curve (**B**) of the PA gradient concentration.

To investigate the specific response of the sensor to target molecules, different concentrations of PA, from 40 μg/mL to 0.1 μg/mL, were prepared for an antibody–antigen interaction. As shown in Figure 5A, after being injected into the flow cell, the peanut allergen specifically bound to the sensor, leading to an increased SPR value of 0.012°. As the PA concentrations rose, the sample response shifts increased gradually from 0.012° to 0.114°, which meets the regulation of direct immunoassay. A standard curve was obtained by fitting the SPR response values dependent on PA concentrations, presenting a good linear relationship of R^2^ = 0.985 (Figure 5B). Under optimal conditions, the detection of PA exhibits a wide range of 40 μg/mL–0.1 μg/mL. The limit of detection for PA was determined to be 0.054 μg/mL, which indicates a good sensitivity. Meanwhile, the sensor stability was also tested with continuous injections. After being regenerated several times with 10 mM HCl, the baseline signal remained at a similar level, as did the sample response, which demonstrates the high stability of the fabricated sensor.

## 4. Conclusions

In summary, a facile and efficient antifouling sensor was developed based on the synergy of an antibody and phase-transited BSA. Through a simple mixing of BSA and TCEP, PTB could be easily obtained and stably grafted onto surfaces by using a drip immersion approach. The PTB coatings exhibit excellent antifouling performance against nonspecific protein adsorptions. With the immobilization of the antibody, the fouling resistance of sensors was further enhanced. The reason may be attributed to the antifouling capability of the antibody itself. The antibody on the sensor surface contributes to forming a neutrally charged coating and shields the electrostatic adsorption antibody. Furthermore, a simple detection assay for peanut allergens was performed with a high efficiency and good sensing performance. The high stability of the sensor, which was evaluated by continuous injections and regenerations, also proves its feasibility and reusability in future applications. This facile strategy for establishing an antifouling sensor provides new ideas for the other sensing devices.

## Data Availability

Data are contained within the article.

## Supplementary Materials

The following supporting information can be downloaded at: https://www.mdpi.com/article/10.3390/bios13121004/s1, Figure S1: Fluorescence spectrum of BSA and PTB mixed with ThT; Figure S2: Image of the PTB-modified surface stained with Congo red; Figure S3: SEM image of the hole on the PTB coating after being continuously irradiated with high-power electron beams; Figure S4: SPR spectral response of BSA adsorbed on bare Au; Figure S5: SPR spectral response of lysozyme adsorbed on bare Au; and Figure S6: Adsorption capacity of lysozyme dissolved in a more stringent buffer (2 ng/cm^2^).
