# Supplementary material for: Design of a Facile Antifouling Sensor Based on the Synergy between an Antibody and Phase-Transited BSA
Source: Biosensors (Basel). 2023 Nov 29;13(12):1004. doi: 10.3390/bios13121004 (PMC10741890; doi:10.3390/bios13121004)
Supplement: Supplementary file 1 [file biosensors-13-01004-s001.zip › biosensors-2721179-supplementary.pdf]

Supplementary Material

Design of a Facile Antifouling Sensor Based on the Synergy between an Antibody and Phase-Transited BSA

Siqi Wang, Xinru Dong, Jialu Li, Jialei Liu, Yifei Ruan and Yinqiang Xia*

1. Fluorescent experiments

Bovine serum albumin (BSA, 1 mg/mL) was mixed with tris(2-carboxyethyl)phosphine (TCEP, 4 mg/ml) at the ratio of 1:1 in the Phosphate-buffered saline buffer (PBS, 10 mM pH 7.4) and incubated for 1 h for a phase transition reaction to obtain the phase-Transited BSA (PTB solution). 100 μL of Thioflavin T (ThT, 1 mg/mL) was added into BSA and PTB solution for monitoring the fluorescence spectra with an excitation wavelength of 420 nm.

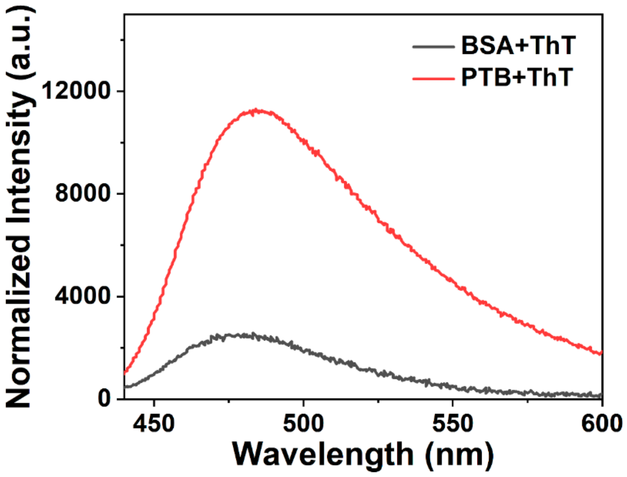

Figure S1. Fluorescence spectrum of BSA and PTB mixed with ThT.

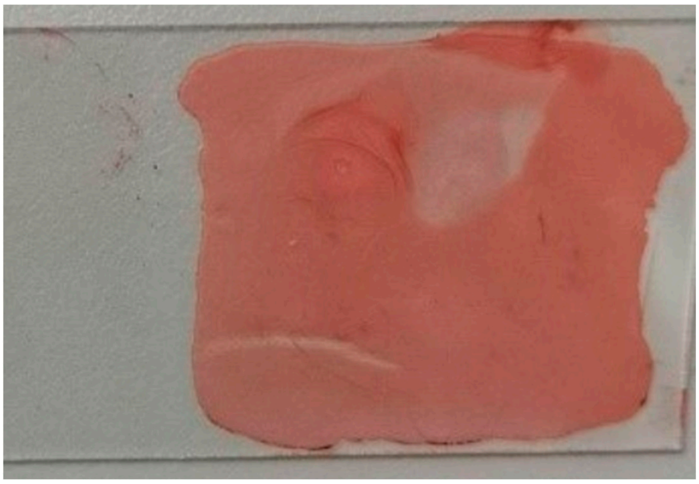

Figure S2. The image of PTB modified surface stained with Congo red.

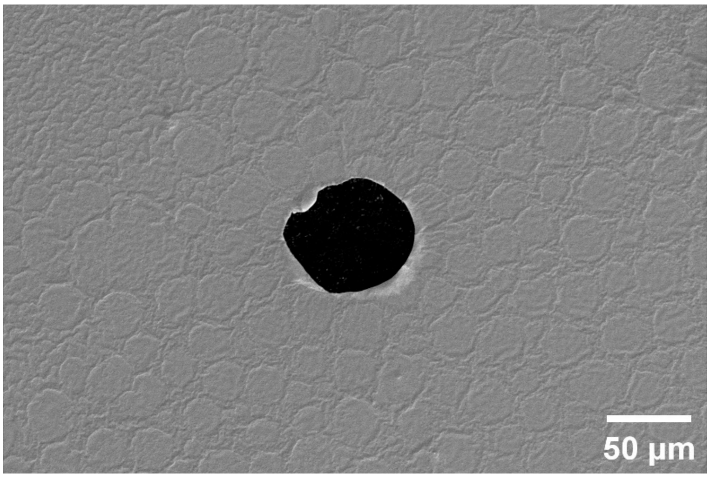

Figure S3. The scanning electron microscopy image of the hole on the PTB coating after being continuously irradiated with high-power electron beams.

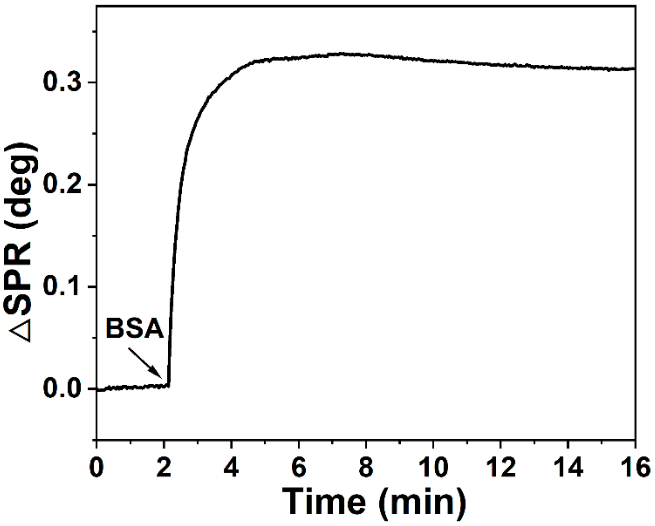

Figure S4. surface plasmon resonance spectral response of BSA adsorbed on bare Au.

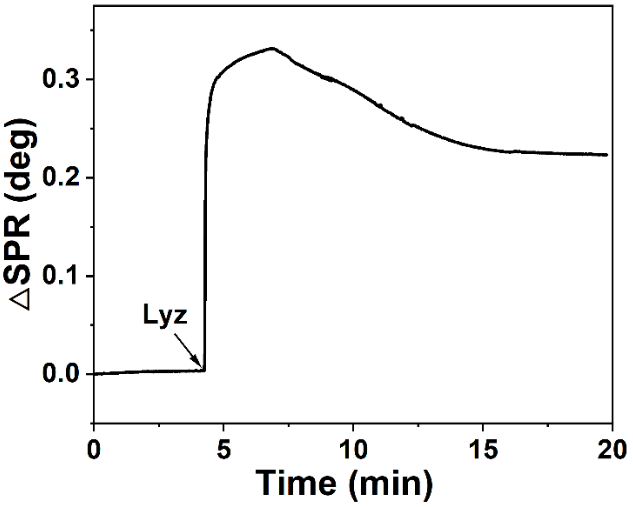

Figure S5. SPR spectral response of Lysozyme adsorbed on bare Au.

biosensors MDPI

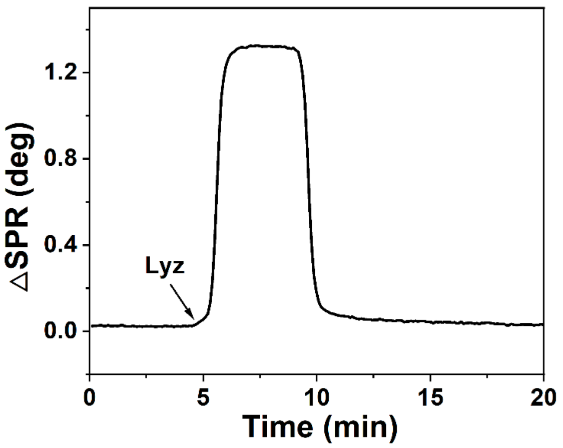

Figure S6. Adsorption capacity of lysozyme dissolved in a more stringent buffer (2 ng/cm^2).
